# Labetalol and soluble endoglin aggravate bile acid retention in mice with ethinylestradiol-induced cholestasis

**DOI:** 10.3389/fphar.2023.1116422

**Published:** 2023-01-26

**Authors:** Ivone Cristina Igreja Sá, Katarina Tripska, Fatemeh Alaei Faradonbeh, Milos Hroch, Hana Lastuvkova, Jolana Schreiberova, Marian Kacerovsky, Miguel Pericacho, Petr Nachtigal, Stanislav Micuda

**Affiliations:** ^1^ Department of Biological and Medical Sciences, Faculty of Pharmacy in Hradec Kralove, Charles University, Hradec Kralove, Czechia; ^2^ Department of Pharmacology, Faculty of Medicine in Hradec Kralove, Charles University, Hradec Kralove, Czechia; ^3^ Department of Biochemistry, Faculty of Medicine in Hradec Kralove, Charles University, Hradec Kralove, Czechia; ^4^ Department of Obstetrics and Gynecology, University Hospital Hradec Kralove, Faculty of Medicine in Hradec Kralove, Charles University, Hradec Kralove, Czechia; ^5^ Biomedical Research Institute of Salamanca and Renal and Cardiovascular Physiopathology Unit, Department of Physiology and Pharmacology, University of Salamanca, Salamanca, Spain

**Keywords:** labetalol, soluble endoglin, ethinylestradiol (EE2), cholestasis, bile acids

## Abstract

Labetalol is used for the therapy of hypertension in preeclampsia. Preeclampsia is characterized by high soluble endoglin (sEng) concentration in plasma and coincides with intrahepatic cholestasis during pregnancy (ICP), which threatens the fetus with the toxicity of cumulating bile acids (BA). Therefore, we hypothesized that both labetalol and increased sEng levels worsen BA cumulation in estrogen-induced cholestasis. C57BL/6J, transgenic mice overexpressing human sEng, and their wild-type littermates were administrated with ethinylestradiol (EE, 10 mg/kg s.c., the mice model of ICP) and labetalol (10 mg/kg s.c.) for 5 days with sample collection and analysis. Plasma was also taken from healthy pregnant women and patients with ICP. Administration of labetalol to mice with EE cholestasis aggravated the increase in BA plasma concentrations by induction of hepatic Mrp4 efflux transporter. Labetalol potentiated the increment of sEng plasma levels induced by estrogen. Increased plasma levels of sEng were also observed in patients with ICP. Moreover, increased plasma levels of human sEng in transgenic mice aggravated estrogen-induced cholestasis in labetalol-treated mice and increased BA concentration in plasma *via* enhanced reabsorption of BAs in the ileum due to the upregulation of the Asbt transporter. In conclusion, we demonstrated that labetalol increases plasma concentrations of BAs in estrogen-induced cholestasis, and sEng aggravates this retention. Importantly, increased sEng levels in experimental and clinical forms of ICPs might present a novel mechanism explaining the coincidence of ICP with preeclampsia. Our data encourage BA monitoring in the plasma of pregnant women with preeclampsia and labetalol therapy.

## 1 Introduction

Bile acids (BAs) have a well-known role in aid of the digestion process by promoting intestinal absorption of dietary lipids. In addition to this classical role, BAs can also function as metabolic hormones regulating glucose and triglyceride metabolism (Trauner and Boyer, 2003). However, when the bile flow is disrupted during cholestasis, BAs can accumulate in plasma, which may impose significant systemic toxicity. Most strikingly, extrahepatic toxicity of BAs is apparent during intrahepatic cholestasis in pregnancy (ICP), where they can produce serious fetal injuries, including stillbirth (Heikkinen et al., 1981; Glantz et al., 2004; Williamson and Geenes, 2014). ICP typically develops in women with preexisting clinically silent impairment of liver BA transporters with manifestation upon the increased level of gonadal hormones such as estrogens (Pascual et al., 2002). Identification of factors that may contribute to increased sensitivity of the liver to the development of ICP is therefore highly desirable.

Endoglin (CD 105) is a transmembrane glycoprotein expressed in the liver at the plasma membrane of endothelial cells, hepatic stellate cells, and Kupffer cells, but not in hepatocytes that act as a coreceptor for members of the TGF-β superfamily (Lopez-Novoa and Bernabeu, 2010; Meurer et al., 2011; Vicen et al., 2021). MMP14 (matrix metalloproteinase 14)-mediated shedding of membrane-bounded endoglin (Eng) leads to the release of the soluble form of endoglin (sEng) into the circulation (Hawinkels et al., 2010). Importantly, our previous studies found that sEng may markedly alter BA metabolomics in healthy mice and mice with non-alcoholic steatohepatitis (Dolezelova et al., 2019; Igreja Sá et al., 2020). Thus, we suggested that sEng could potentially play a negative role in the pathogenesis of various liver disorders.

It is crucial to mention that sEng is an important biomarker of preeclampsia, a disease characterized by hypertension, proteinuria, and the risk of serious (even fatal) health problems for the mother and fetus (Venkatesha et al., 2006). Additionally, sEng was demonstrated to induce arterial hypertension *via* Bone morphogenetic protein 4 (BMP4) (Gallardo-Vara et al., 2020), and high plasma sEng levels are associated with preeclampsia symptoms and placental abnormalities, suggesting the crucial role of sEng in preeclampsia development (Pérez-Roque et al., 2020).

Hypertension during preeclampsia is considered a significant danger to both mother and fetus that requires prompt treatment. In clinical practice, hypertension during preeclampsia with severe features is managed by administering labetalol, an alpha1-and beta-adrenergic receptor antagonist (Lu et al., 2018; Peres et al., 2018). However, labetalol was linked to drug-induced liver damage and ICP in pregnant women (Firoz et al., 2015; Whelan et al., 2020). Interestingly, recent studies have demonstrated a markedly increased incidence of preeclampsia in women with ICP (Raz et al., 2015; Marathe et al., 2017; Liu et al., 2020; Mor et al., 2020). Nevertheless, the cause of the simultaneous worsening of liver functions, labetalol treatment, and possible involvement of sEng in these processes is unknown.

Therefore, in this study, we hypothesized that ethinylestradiol-induced cholestasis increases sEng levels, and labetalol treatment in this situation aggravates the cumulation of BA in plasma. Additionally, we hypothesized that high sEng levels precipitate a further increase of BA plasma concentrations in labetalol-treated cholestatic mice.

## 2 Materials and methods

### 2.1 Chemicals

Ethinylestradiol (EE) and labetalol (L) (>98% purity) were purchased from Merck (Darmstadt, Germany). Bile acid standards were purchased from Steraloids, Inc. (Newport, Rhode Island, United States) and Sigma-Aldrich (St Louis, Missouri, United States).

### 2.2 Animals and experimental design

Animals were housed under a 12-h light cycle and constant temperature (22°C ± 1°C) and humidity and had free access to respective drink and pellet diet. All institutional and national guidelines for the care and use of laboratory animals were followed. The Ethical Committee for the Protection of Animals Against Cruelty at the Faculty of Pharmacy, Charles University (Permit Number 4937/2019-8), and the Bioethics Committee of the University of Salamanca (Permit Number: 006–201400038812) approved all animal procedures applied in the project. All efforts were made to minimize the suffering of the animals.

Eight-week-old female C57BL/6J mice were purchased from Velaz (Prague, Czech Republic). After a 2-week adaptation period, the mice were administrated daily, for 5 days, subcutaneously (s.c.) with propanediol as the control group (C), ethinylestradiol (10 mg/kg s.c.) (EE) to induce intrahepatic cholestasis, labetalol (10 mg/kg s.c.) (L) or concomitant administration of ethinylestradiol (10 mg/kg) with labetalol (10 mg/kg s.c.) (EEL) simulating the antihypertensive treatment during intrahepatic cholestasis in pregnancy. Indeed, ethinylestradiol (the major estrogen sex hormone in humans and a widely used medication) is used for estrogen-induced cholestasis, an animal model for intrahepatic cholestasis of pregnancy (Muchova et al., 2015; Faradonbeh et al., 2021; Gijbels et al., 2021).

To assess the effect of increased levels of sEng in cholestasis treated by labetalol, we used ten-week-old female transgenic mice overexpressing human soluble endoglin (hsEng) on the CBAxC57BL/6J background generated at the Genetically Modified Organisms Generation Unit (University of Salamanca, Spain), an useful animal model for research in preeclampsia (Valbuena-Diez et al., 2012; Pérez-Roque et al., 2020). Their wild-type littermates (WT) were used as the control group. Both groups of mice were administrated daily, for 5 days, subcutaneously (s.c.) with the association of ethinylestradiol (10 mg/kg) and labetalol (10 mg/kg). The selected labetalol dose was based on the clinically used dose regimen, with respect to interspecies differences, and on the dosage used in other studies (Tritapepe et al., 1980; Kubota et al., 1990; Meng et al., 2015; Kim et al., 2018). Administration (both ethinylestradiol and labetalol) was performed in short-term anesthesia induced by isoflurane. After the last administration, mice were placed in metabolic cages for stool collection for 24 h.

On day 6, mice were fasted for 10 h and then anesthetized with pentobarbital (50 mg/kg, i.p.), and bile was collected for 45 min from the cannulated gallbladder. Blood was collected from the *inferior vena cava*. Animals were then sacrificed by anesthetic overdose, followed by liver tissue and ileum harvest for biochemical and molecular analyses. As previously described, plasma samples were obtained from the whole blood by centrifugation (Igreja Sa et al., 2020). Samples were stored at −80°C until analysis.

### 2.3 Analytical methods

Plasma concentration of liver enzymes and bilirubin were determined using a commercial Preventive Care Profile Plus test and Vetscan 2 device (Abaxis, United States), as previously (Igreja Sa et al., 2020). Analysis of endogenous BA content in plasma, bile, and feces was performed by liquid chromatography coupled with mass spectrometry (LC-MS) as described previously (Dolezelova et al., 2019; Igreja Sa et al., 2020).

### 2.4 Quantitative real-time RT-PCR and western blot analysis

mRNA expression was analyzed by reverse transcription-polymerase chain reaction (qRT-PCR) in QuantStudio™ 7 Flex Real-Time PCR System (Applied Biosystems, Foster City, United States). The Glyceraldehyde 3-phosphate dehydrogenase (Gapdh) gene was used as a reference for normalizing data. Primers used for analysis are specified in Supplementary Table S1.

For Western blot analysis, the total fraction and crude plasma membrane fraction were prepared from mice liver homogenates as described previously (Prasnicka et al., 2017). Proteins (25 µg) were separated by SDS–PAGE, transferred to the PVDF membrane (Millipore, New York, NY, United States), and incubated with appropriate antibodies (Supplementary Table S2). The chemiluminescence image of bands was captured using Evolution-capt software (Fusion Solo 6S Edge, Vilber Lourmat SAS, France) and quantified using ImageLab imaging software version 6.0.1 (Bio-Rad). The immunodetection of Gapdh confirmed the equal loading of proteins onto the gel.

### 2.5 Enzyme-linked immunosorbent assay

The concentration of hsEng present in mice and human plasma was determined using a Human Endoglin/CD105 Quantikine ELISA kit (DNDG00, R&D Systems, Minneapolis, MN, United States) according to the manufacturers’ instructions in duplicates. Transgenic mice express variable levels of human soluble endoglin (hsEng), so mice expressing plasma levels of hsEng above the 500 ng/mL threshold were selected. On the other hand, their wild-type (WT) littermates presented undetectable hsEng levels in plasma.

Plasma levels of mouse sEng (msEng) levels were determined by Mouse Endoglin/CD105 Quantikine (MNDG00, R&D Systems, Minneapolis, MN, United States) ELISA Kit, used according to the manufacturer’s instructions, in duplicates.

### 2.6 Patients and samples

This retrospective study included pregnant women admitted to the Department of Obstetrics and Gynecology of the University Hospital Hradec Králové, Czech Republic, between February 2019 and May 2020. Thirteen pregnant women with singleton pregnancies were diagnosed with ICP (pruritus and an elevation in plasma bile acids levels), and ten healthy pregnant women with uncomplicated singleton pregnancies were recruited. Women younger than 18 years and women with pregnancy-related and other medical complications, such as fetal growth restriction, congenital or chromosomal fetal abnormalities, gestational or pregestational diabetes, chronic or gestational hypertension, preeclampsia, or significant vaginal bleeding, were excluded. None of the women received antihypertensive drugs. The samples of maternal plasma were obtained. The gestational week of the sampling was the third trimester (>28 weeks). Gestational age was determined based on first-trimester fetal biometry. All participants in the study were Caucasian. This study was approved by the Institutional Review Board Committee (7 February 2019; No. 201902 S16P). All procedures followed were in accordance with the ethical standards of the responsible committee on human experimentation (institutional and national) and with the Helsinki Declaration of 1975, as revised in 2000. Informed consent was obtained from all patients included in the study.

### 2.7 Statistical analysis

All analyses were performed using GraphPad Prism 8.0 software (San Diego, California, United States). The data are presented as mean ± SEM. The normal distribution of the data was confirmed by Shapiro–Wilk test (Mishra et al., 2019). Comparison between all groups to control was carried out using One-way ANOVA, and direct comparisons of EE—EEL and WT—hsEng were carried out using an unpaired *t*-test. A difference of *p* < 0.05 was considered statistically significant.

## 3 Results

### 3.1 Labetalol treatment aggravates ethinylestradiol-induced cholestasis

**FIGURE 1:**
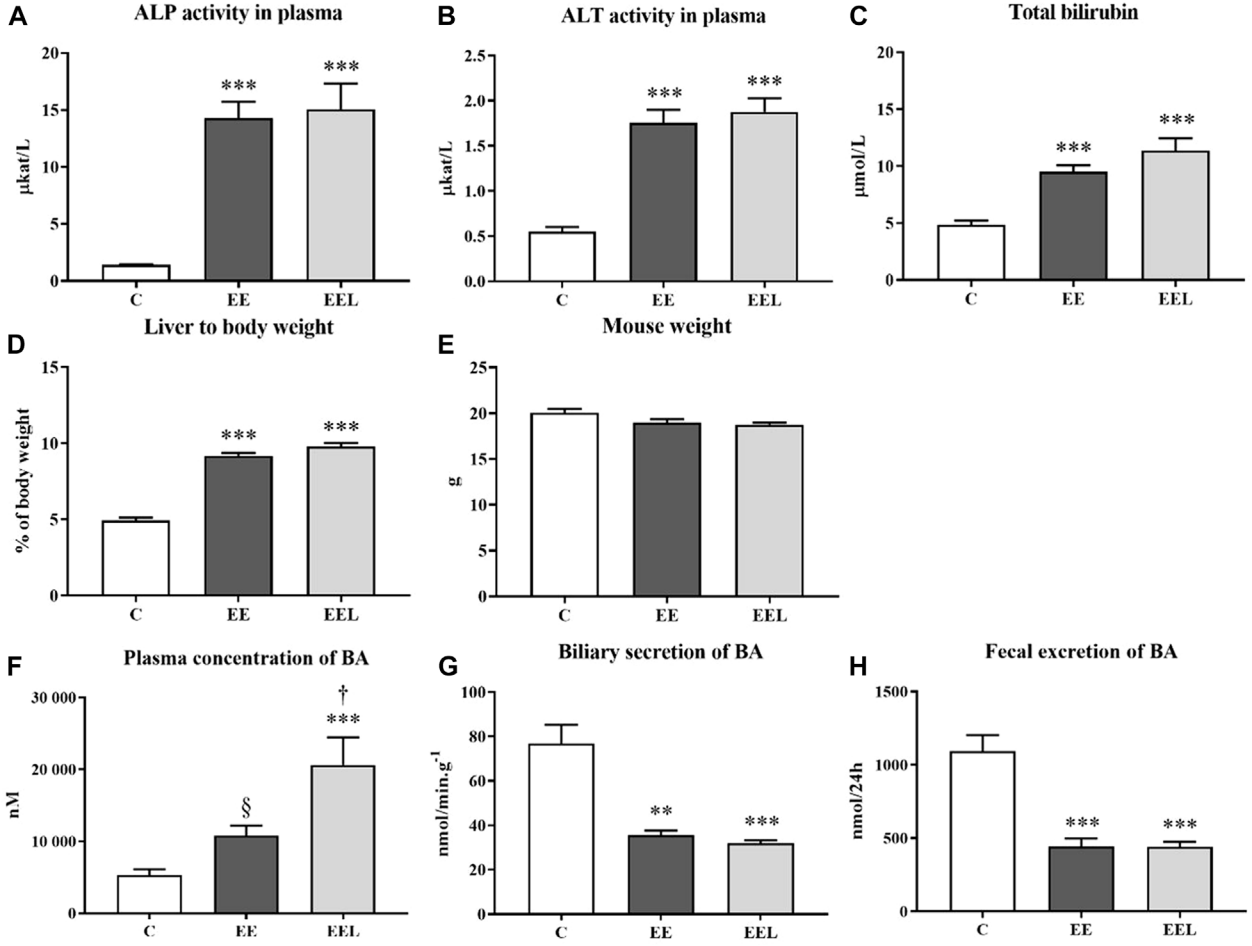
Ethinylestradiol and labetalol effects on parameters of liver injury. The activity of ALP **(A)** and ALT **(B)** and bilirubin levels **(C)** in plasma. Ratio liver to body weight **(D)** and mouse body weight **(E)**. Plasma concentration of BAs **(F)**. Biliary elimination of BAs **(G)** and fecal excretion of BAs **(H)**. The data are presented as median with SEM (*n* = 8). **p < 0.01, ***p < 0.001, by One-Way ANOVA test comparing vehicle-administered control group with cholestatic groups. ^†^ *p* < 0.05, ^§^ *p* = 0.07 using the unpaired *t*-test for cholestatic animals (EE) vs. cholestatic animals treated with labetalol (EEL).

Administration of labetalol in control healthy animals (L) did not result in significant changes in any biochemical parameters in comparison to the vehicle-administrated controls (C) (Supplementary Figure S1). However, EE induced a typical liver injury characterized by significantly increased: plasma activity of liver enzymes ALP and ALT (Figures 1A, B), plasma concentrations of total bilirubin (Figure 1C), and liver to-body weight ratio (Figure 1D) despite no change in mouse body weight compared with untreated control mice (Figure 1E). Estrogen-induced cholestasis was confirmed by significantly increased concentrations of BAs in plasma (Figure 1F) and reduced biliary and fecal excretion of BAs (Figures 1G, H). These data are in accordance with the previously published animal model of ethinylestradiol-induced cholestasis (Faradonbeh et al., 2021).

### 3.2 Labetalol increased BA levels in plasma are mediated by increased expression of Mrp4 efflux transporter

**FIGURE 2:**
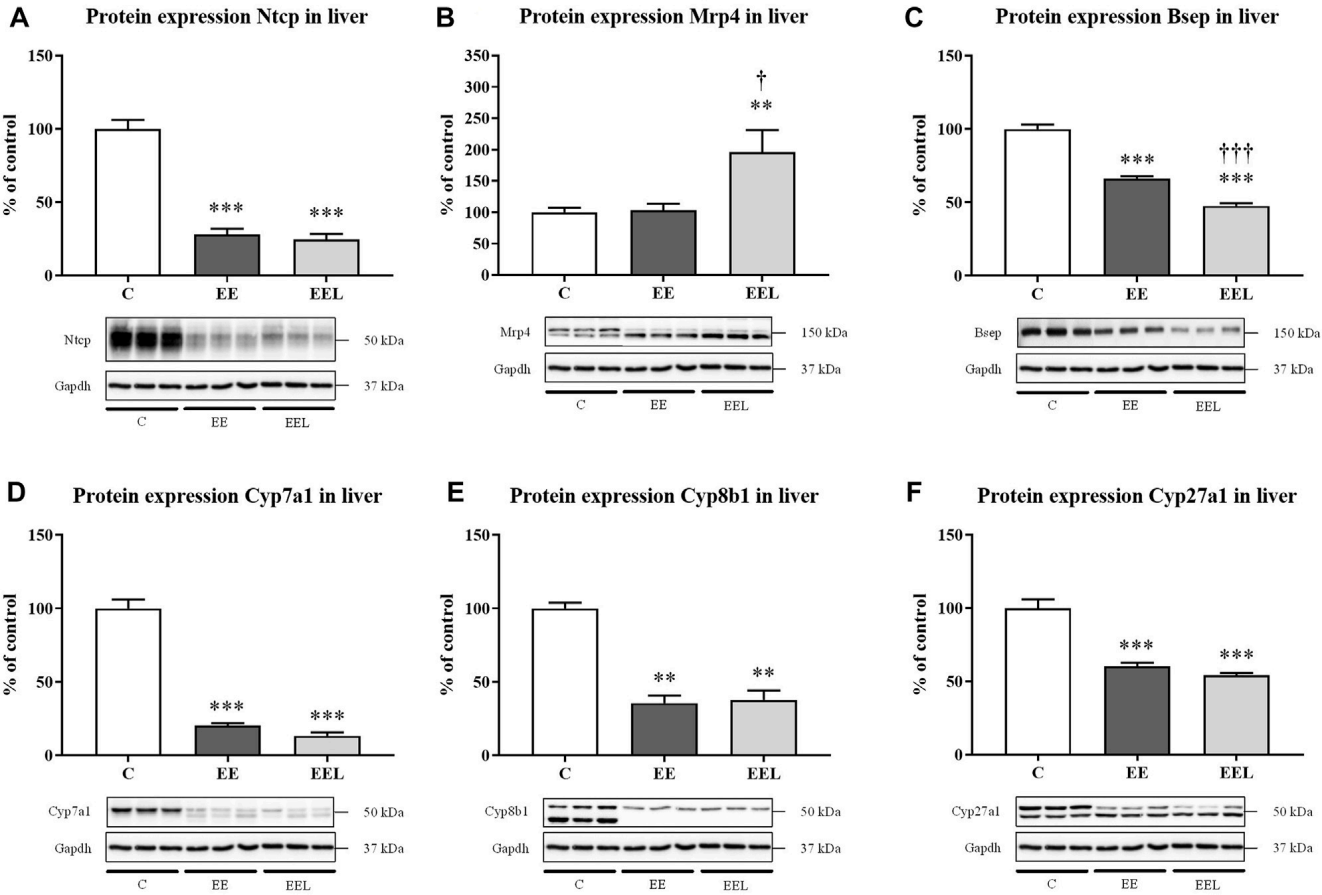
Labetalol affects BA levels in plasma by modulation of BA basolateral Mrp4 transporter. Protein expression of BA transporters Ntcp **(A)**, Mrp4 **(B)**, and Bsep **(C)** in the liver. Protein expression of BA synthesizing enzymes Cyp7a1 **(D)**, Cyp8b1 **(E)**, and Cyp27a1 **(F)**. The data are presented as median with SEM (*n* = 8). **p < 0.01, ***p < 0.001, by One-Way ANOVA test comparing vehicle-administered control group with cholestatic groups. ^†^ *p* < 0.05, ^†††^ *p* < 0.001 using the unpaired *t*-test for cholestatic animals (EE) vs. cholestatic animals treated with labetalol (EEL). The images of loading control (Gapdh) are re-used for illustrative purposes.

Determination of the mechanism of increased BA plasma concentrations in labetalol-treated mice with ethinylestradiol-induced cholestasis (Figure 1F). We evaluated the liver protein expression of the BA-related transporters (Ntcp, Mrp4, and Bsep) and enzymes (Cyp7a1, Cyp8b1, and Cyp27a1). Untreated EE mice predictably developed the reduced protein expression of Ntcp (Figure 2A), Bsep (Figure 2C), Cyp7a1 (Figure 2D), Cyp8b1 (Figure 2E), and Cyp27a1 (Figure 2F). Interestingly, labetalol administration to cholestatic mice did not modulate Ntcp and Cyps expressions (Figure 2A), but it significantly increased the protein expression of Mrp4 (Figure 2B) and downregulated Bsep (Figure 2C). Nrf2 signaling is involved in the regulation of Mrp4. Therefore, we analyzed mRNA of Nrf2-target genes such as *Nqo1* (*NAD*(*P*)*H quinone dehydrogenase 1*), *Gpx2 (glutathione peroxidase 2)*, and *Gclm (glutamate-cysteine ligase modifier subunit)*. Labetalol administration did not change the expression of *Nqo1* and *Gclm* genes but induced *Gpx2* mRNA expression (Supplementary Figure S2). Absence of consistent results indicates that Nrf2 was not responsible for the observed changes in the Mrp4 protein.

### 3.3 Ethinylestradiol-induced cholestasis and labetalol increase sEng levels in plasma

**FIGURE 3:**
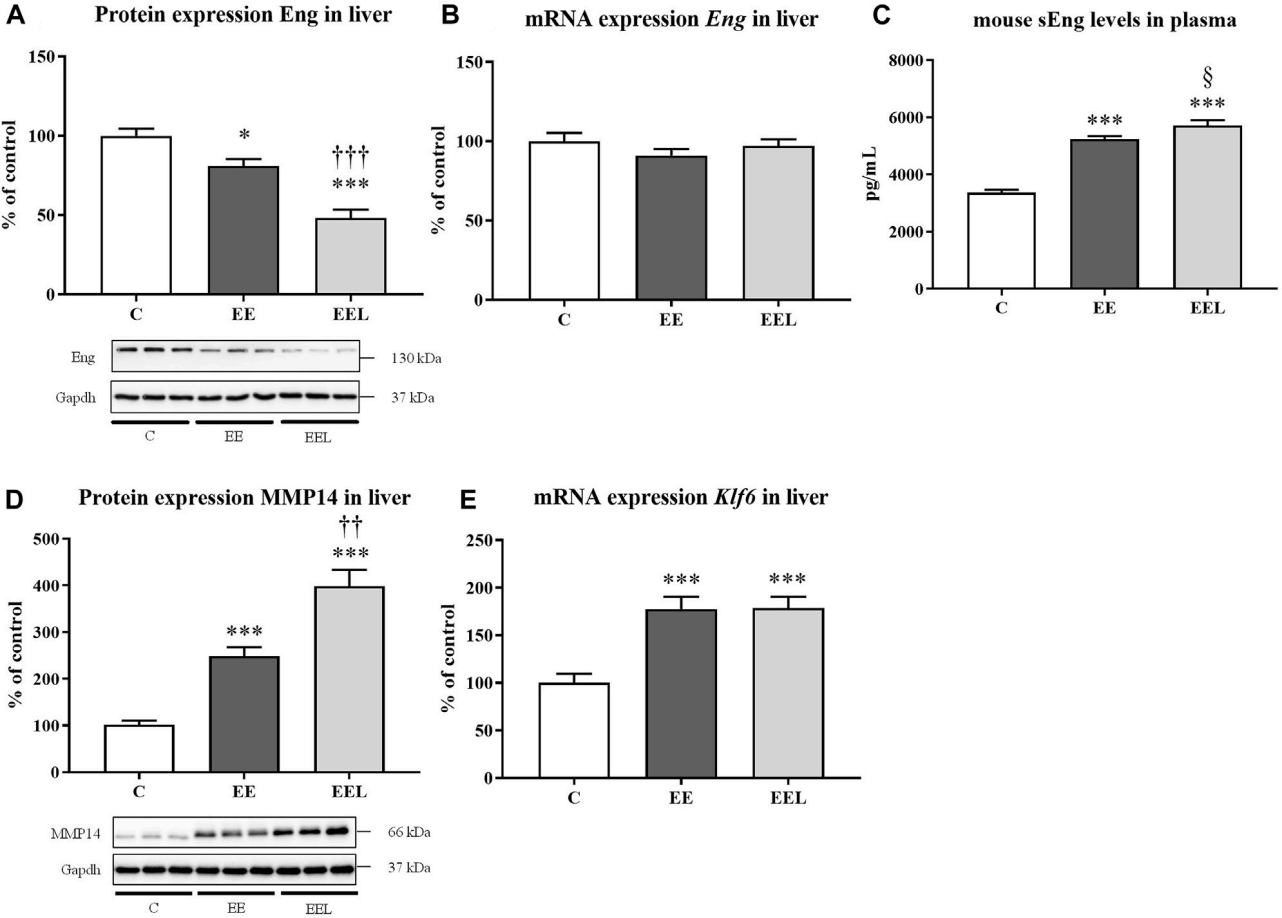
Ethinylestradiol and labetalol effects on endoglin homeostasis. Protein and mRNA expression of Eng in the liver **(A, B)**. Plasma levels of sEng **(C)** and protein levels of MMP14 **(D)**. The mRNA expression of transcription factor *Klf6* **(E)**. The data are presented as median with SEM (*n* = 8). *p < 0.05, ***p < 0.001, by One-Way ANOVA test comparing vehicle-administered control group with cholestatic groups. ^††^ *p* < 0.05, ^†††^ *p* < 0.01, ^§^ *p* = 0.059 using the unpaired *t*-test for cholestatic animals (EE) vs. cholestatic animals treated with labetalol (EEL).

Administration of labetalol in healthy control animals did not result in significant modulation of the Eng pathway when compared to untreated healthy mice (Supplementary Figure S3). Administration of EE to mice resulted in reduced protein expression of Eng (Figure 3A) despite no significant change in mRNA levels (Figure 3B). In addition, EE treatment increased levels of mouse sEng in plasma compared to control mice (Figure 3C). EE animals also showed increased protein expression of MMP14 (Figure 3D), as well as increased mRNA expression of *Klf6*. Labetalol treatment of cholestatic mice resulted in a further increase in protein expression of MMP14 and sEng levels (*p* = 0.059) in association with significantly reduced protein expression of Eng when compared to untreated cholestatic mice (Figure 3).

### 3.4 sEng levels are increased in pregnant women with intrahepatic cholestasis

**FIGURE 4:**
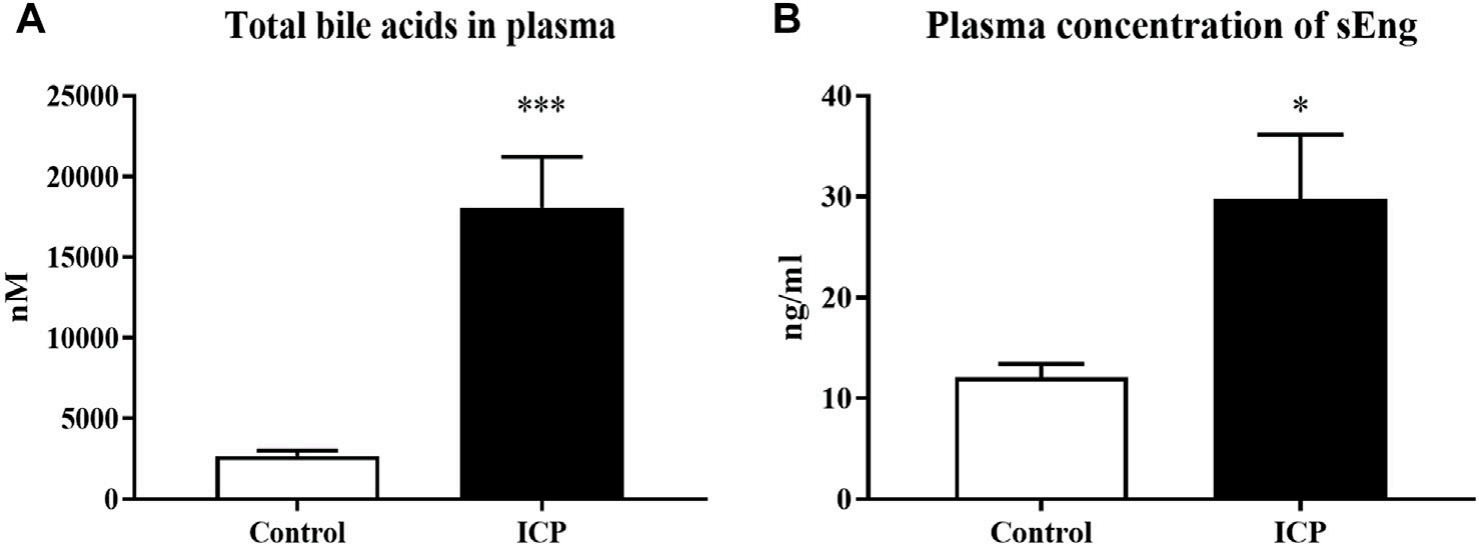
sEng levels are increased in patients with intrahepatic cholestasis of pregnancy. Total bile acid concentration in plasma **(A)**. Plasma levels of sEng **(B)**. The data are presented as mean with SEM. *p < 0.05, ***p < 0.001, by Mann-Whitney test.

Plasma samples from pregnant women diagnosed with intrahepatic cholestasis and healthy pregnant donors were analyzed. Results showed a significantly increased concentration of BAs (Figure 4A) and sEng (Figure 4B) in the plasma of the ICP-diagnosed patients in comparison to healthy pregnant controls.

### 3.5 sEng aggravates cholestasis in labetalol-treated mice with estrogen-induced cholestasis

**FIGURE 5:**
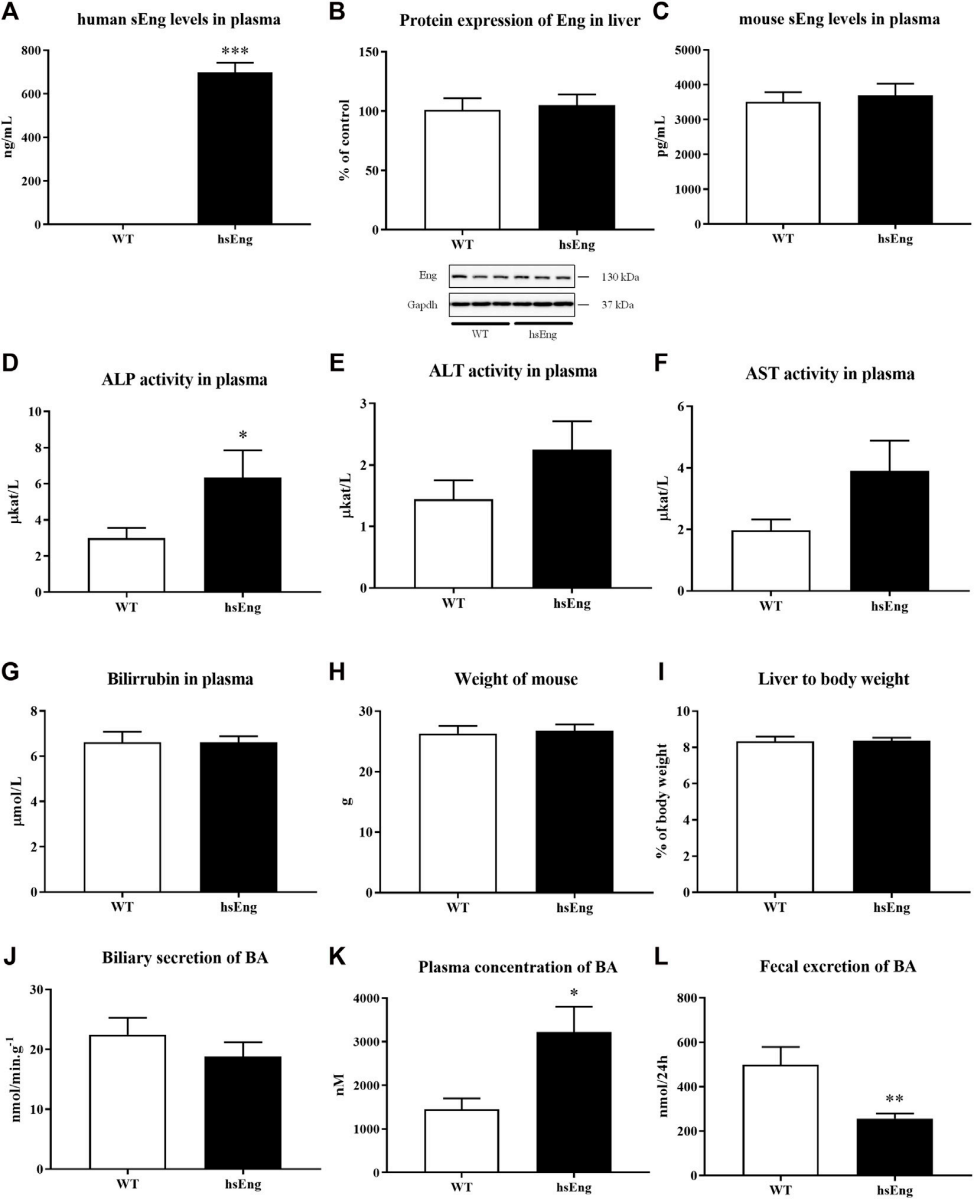
High levels of human hsEng in cholestatic animals treated with labetalol resulted in increased plasma concentration of BA. Plasma levels of human sEng **(A)**, protein expression of Eng in the liver **(B)**, and plasma levels of mouse sEng **(C)**. Plasma activities of liver enzymes ALP **(D)**, ALT **(E)**, and AST **(F)**. Bilirubin levels in plasma **(G)**. Mouse weight **(H)** and ratio liver to body weight **(I)**. Biliary secretion of BAs **(J)**, plasma concentration **(K)**, and fecal excretion **(L)** of BAs. The data are presented as median with SEM (*n* = 8). *p < 0.05, **p < 0.01, ***p < 0.001, by unpaired *t*-test.

The hsEng mice present high hsEng levels in their plasma (Figure 5A) compared to their WT littermates, where human sEng is not detectable. Estrogen-induced cholestasis and labetalol treatment were used in both WT and hsEng groups. The results showed that increased levels in hsEng do not significantly modulate the expression of mouse Eng (Figure 5B) nor mouse sEng levels in their plasma (Figure 5C). On the other hand, increased levels of hsEng resulted in worsening of liver damage, as demonstrated by the significant increase in plasma activities of ALP (Figure 5D), while ALT (Figure 5E) and AST (Figure 5F) were not affected. No significant difference was observed in total bilirubin levels in plasma (Figure 5G), mouse weight (Figure 5H), liver-to-body weight ratio (Figure 5I), and biliary secretion of BAs (Figure 5J) when compared hsEng mice to WT animals. However, high hsEng levels resulted in an increased concentration of circulating BAs in plasma (Figure 5K) and reduced fecal excretion (Figure 5L).

### 3.6 sEng increases the reabsorption of BA into entero-hepatic circulation

**FIGURE 6:**
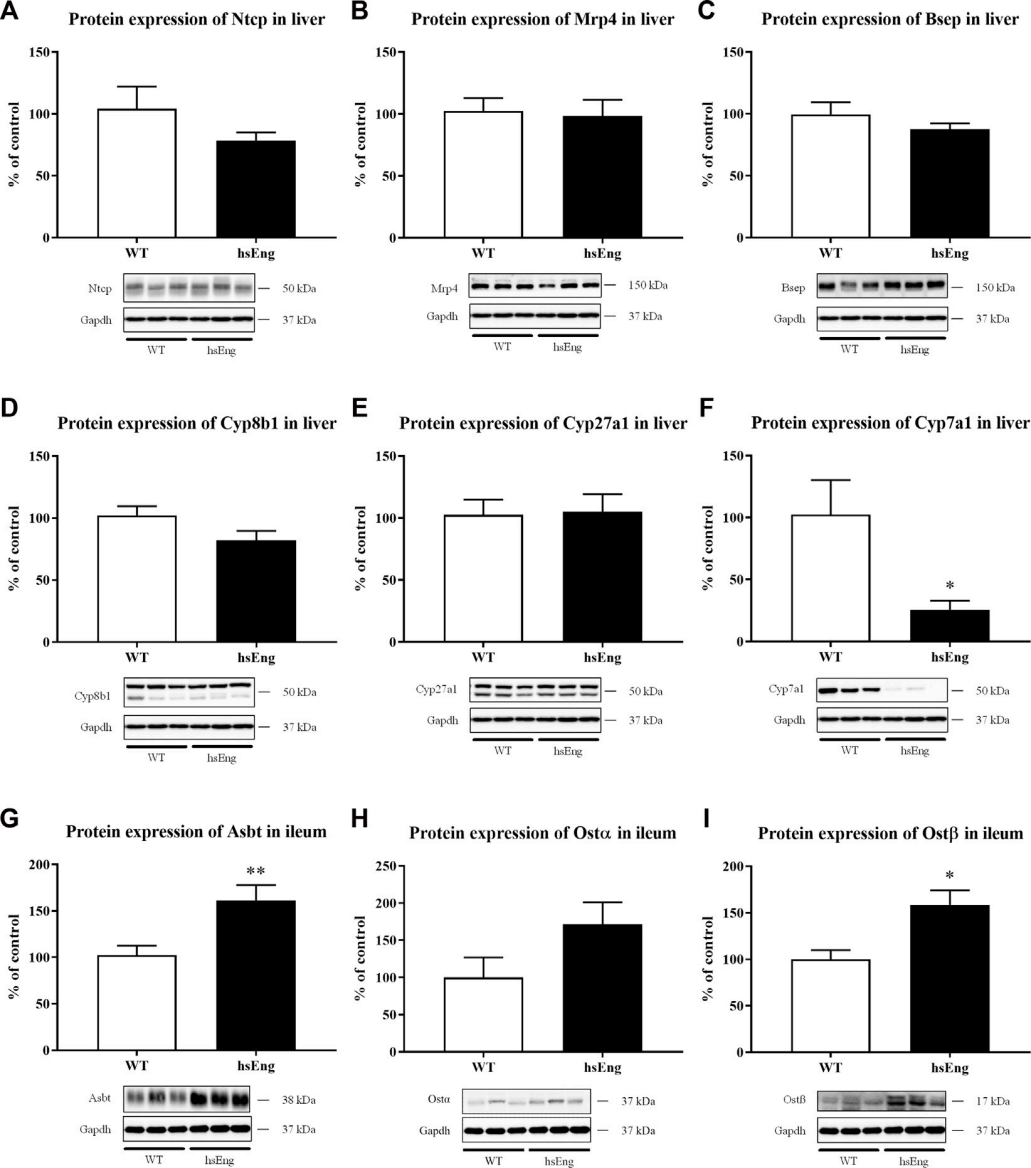
Increased plasma concentration of BAs is due to increased enterohepatic reabsorption of BA. Protein expression of basolateral hepatic transporters Ntcp **(A)** and Mrp4 **(B)**. Protein expression of canalicular hepatic transporter, Bsep **(C)**. Protein expression of BA synthesizing enzymes Cyp8b1 **(D)**, Cyp27a1 **(E)**, and Cyp7a1 **(F)**. Protein expression of ileal transporters Asbt **(G)**, Ostα **(H)**, and Ostβ **(I)**. The data are presented as median with SEM (*n* = 8). *p < 0.05, **p < 0.01, by unpaired *t*-test. The images of loading control (Gapdh) are re-used for illustrative purposes.

No significant difference in the hepatic protein expression of Ntcp (Figure 6A), Mrp4 (Figure 6B), Bsep (Figure 6C), Cyp8b1 (Figure 6D), and Cyp27a1 (Figure 6E) when comparing with labetalol and ethinylestradiol-treated hsEng mice with their WT littermates, were demonstrated. On the other hand, we showed significant downregulation of hepatic Cyp7a1 (Figure 6F) and upregulation of ileum Asbt (apical sodium-dependent bile acid transporter) (Figure 6G) as well as Ostα/β (Figures 6H, I) in hsEng mice.

## 4 Discussion

Increased BA concentrations in plasma are the major pathological factor inducing fetal injuries in mothers with ICP. Due to the increased coincidence of ICP with preeclampsia (Raz et al., 2015; Marathe et al., 2017; Mor et al., 2020), there is an increased chance that antihypertensive therapy with labetalol will be used during this form of cholestasis. However, labetalol was previously linked to drug-induced liver damage in pregnant women (Firoz et al., 2015; Whelan et al., 2020). Therefore, we evaluated the impact of labetalol treatment on BA turnover in estrogen-induced cholestasis, the animal model for ICP. The principal finding was that labetalol doubled plasma concentrations of BAs in EE-treated mice, reaching the average concentration of 20 μM. Concentrations of BAs of 20 and more μmol/L in the plasma of pregnant women is a diagnostic indicator for ICP, and clinical guidelines recommend initiation of pharmacotherapy because the status significantly increases the probability of fetal complications such as spontaneous preterm deliveries, asphyxia events, and meconium staining of amniotic fluid, placenta, and membranes (Glantz et al., 2004). Thus, we suggest that labetalol may significantly aggravate the estrogen-induced BA retention in plasma, enhancing the systemic toxicity of these compounds. Although we did not detect worsening of other biochemical indicators of liver injury by labetalol, leaving the increased BA concentrations unresolved may precipitate more serious liver injury over long-term treatment, which is supported by a higher incidence of liver diseases such as hepatitis C or cholangitis in women with ICP (Ropponen et al., 2006).

The plasma concentrations of BAs are increased during ICP and other forms of estrogen-related cholestasis as the consequence of their impaired biliary secretion due to alterations in involved BA transporting proteins (Anwer, 2004; Appelman et al., 2021; Faradonbeh et al., 2021). This effect was confirmed in our vehicle-administered cholestatic group, which showed markedly reduced biliary secretion of BAs in response to the downregulation of Ntcp, the major transporter for uptake of BAs from portal blood into hepatocytes, and Bsep, the rate-limiting transporter in BA biliary secretion. A proportional decrease in fecal excretion of BAs suggested that intestinal reabsorption of BAs, which accounts for at least 95% of their input to the lumen of duodenum *via* the bile duct, was not altered in these animals. Surprisingly, labetalol did not modify estrogen-induced changes in BA biliary and fecal excretion, indicating that increased BA concentrations in the plasma of this group resulted from the impaired flux of BAs through the basolateral membrane of hepatocytes.

In agreement, we detected upregulation of Mrp4, a basolateral transporter for the efflux of BAs from hepatocyte to blood (Trauner and Boyer, 2003), underscoring the return of BAs from hepatocytes to blood as the major mechanism of increased plasma BAs by labetalol. Indeed, previous studies have shown that upregulated expression of Mrp4 at the basolateral membrane seems to be responsible for the high circulating BA levels during a cholestatic injury in rodents and pediatric patients with progressive familial intrahepatic cholestasis (Keitel et al., 2005; Miura et al., 2011). Moreover, Mennone et al. showed reduced BA levels in the circulation of Mrp4 knockout mice than in wild-type cholestatic mice by common bile duct ligation, indicating a role of murine Mrp4 in the adaptive response to cholestatic liver injury (Mennone et al., 2006). However, labetalol also caused additional downregulation of Bsep in our cholestatic group. It may contribute to reverse BA transport back to plasma by increased intrahepatic BA content. Biliary secretion of BAs may still be maintained by alternative canalicular BA transporter such as Mrp2 (Alaei Faradonbeh et al., 2022), which was not changed by labetalol (unpublished observation). Taken together, we suggest that the upregulation of Mrp4 is the principal mechanism of increased BA plasma concentrations by labetalol in the estrogen model of ICP.

The cause of the increased coincidence of ICP and preeclampsia is not known (Raz et al., 2015; Marathe et al., 2017; Liu et al., 2020; Mor et al., 2020). However, it was demonstrated that the total incidence of preeclampsia was not only significantly higher for the patients with ICP, but also the severity of ICP is a major risk factor for preeclampsia development (Raz et al., 2015). Interestingly, preeclampsia usually occurs within 2–4 weeks after the diagnosis of ICP (Raz et al., 2015). It indicates that ICP triggers pathological mechanisms promoting preeclampsia. The major factor connected with the pathology and diagnosis of preeclampsia is increased plasma concentration of sEng (Gallardo-Vara et al., 2020; Pérez-Roque et al., 2020). Therefore, we measured sEng plasma concentrations in women with ICP and in mice administered with estrogen, respectively. Both these measurements showed an association between increased concentrations of BAs and sEng in plasma. These data together indicate that the increased sEng in plasma may be the pathophysiological mechanism connecting the increased coincidence of preeclampsia with ICP.

Therefore, we evaluated a possible mechanism employed in sEng regulation by estrogen in mice liver. Our results indicate that ethinylestradiol increased levels of mouse sEng in plasma *via* increased mRNA expression of *Klf6*, the transcription factor regulating MMP14. Consequent upregulation of the MMP14 protein, the principal cleavage molecule for Eng (Lin et al., 2020), subsequently released sEng to circulation and reduced Eng tissue expression. An identical pattern of sEng regulation was observed in another study with human colorectal carcinoma (Hawinkels et al., 2010). Interestingly, when cholestatic mice were treated with labetalol, a further increase in protein expression of MMP14, sEng levels, and reduced protein expression of Eng indicated that other factors, such as an additional increase in BA concentrations rather than estrogen itself, may be behind sEng regulation. Thus, we suggest that circulating levels of sEng raised in our study in parallel with the severity of BA accumulation might represent a novel biomarker of ICP.

Our previous study elucidated that sEng itself can also modulate BA homeostasis in healthy mice, where the presence of high levels of sEng resulted in increased plasma concentrations of BAs (Dolezelova et al., 2019). We hypothesized that labetalol treatment of sensitive preeclamptic women with high sEng levels might provoke the development of ICP and shift preeclampsia toward a more serious form such as HELLP syndrome (hemolysis, elevated liver enzymes, and low platelet count) characterized by liver damage. In agreement, women with HELLP show higher circulating sEng and BA levels when compared to preeclampsia patients (Venkatesha et al., 2006; Jebbink et al., 2015). To establish the impact of increased sEng levels in estrogen-induced cholestasis in mice treated with labetalol, we used transgenic mice overexpressing human soluble endoglin (hsEng), a recognized model of preeclampsia, and their wild-type (WT) littermates, as control.

It is interesting to mention that there is a high level of homology between mouse and human sEng (99% sequence overlap, 69% identity) (Gougos and Letarte, 1990; St-Jacques et al., 1994). Indeed, it was demonstrated that human sEng affected mouse endoglin signaling and aggravated mice aortic endothelial dysfunction (Vitverova et al., 2018), mice cholesterol and BA metabolism (Dolezelova et al., 2019), mice NASH (Igreja Sa et al., 2020) and induced symptoms of preeclampsia in mice (Venkatesha et al., 2006).

**FIGURE 7:**
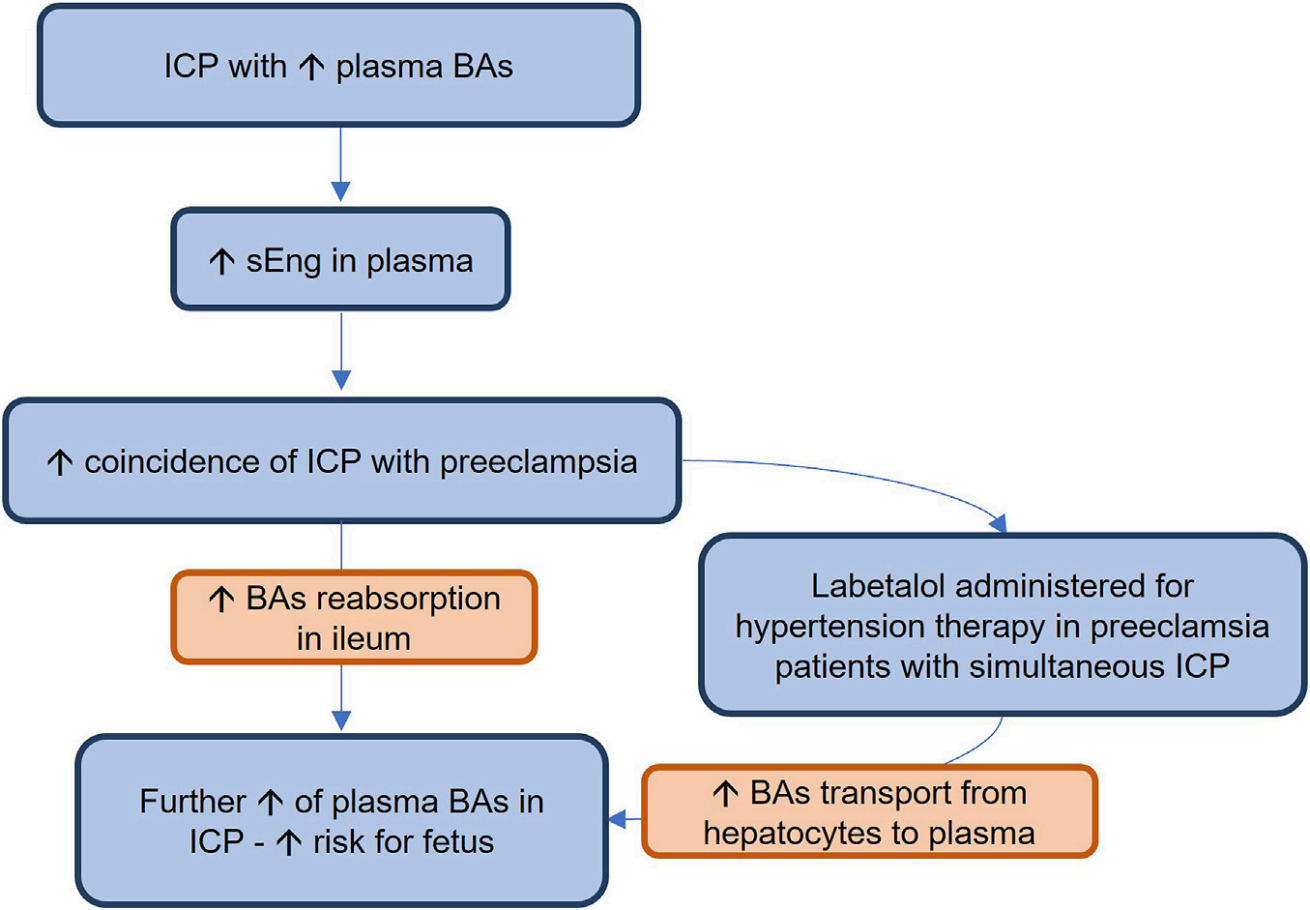
Intrahepatic cholestasis (ICP) is a serious complication of pregnancy, threatening the fetuses with the toxicity of cumulating bile acids (BAs). In this study, we showed for the first time that ICP is accompanied by increased plasma levels of soluble endoglin (sEng). It may explain the increased coincidence of ICP with preeclampsia, a condition characterized by high soluble endoglin (sEng) concentrations. Increased sEng may increase BA plasma concentrations by stimulating their reabsorption in the ileum. In addition, labetalol, when used for therapy of hypertension during preeclampsia in patients with simultaneous ICP, may further increase BA plasma concentrations by enhancing their export from hepatocytes to plasma, increasing the toxicity risks for the fetus.

In this study, we demonstrated that hsEng resulted in aggravation of estrogen + labetalol-induced impairment of BA turnover, as indicated by the significant increase in plasma levels of BAs. The absence of changes in canalicular secretion of BAs and in the expression of major liver BA transporting proteins in the hsEng group, together with the reduction of BA fecal excretion, and downregulation of hepatic Cyp7a1, suggests that increased BA concentrations in plasma of labetalol-treated cholestatic mice resulted from increased intestinal reabsorption rather than from impaired flux of BA through the basolateral/canalicular membrane of hepatocytes or the increased BA synthesis. Indeed, analysis of ileal BA transporters confirmed significantly increased expression of Asbt, an uptake transporter for BA reabsorption at the apical membrane of enterocytes (Yang et al., 2020), as well as Ostα/β, the transporter for export of BAs into the portal circulation at the basolateral membrane of enterocytes in hsEng mice (Ticho et al., 2019). Ileal increase in BA absorption with the subsequent BA overload may contribute to severe systemic and liver toxicity *via* the spillover transport capacity of hepatocyte basolateral uptake transporters (Ticho et al., 2019; Yang et al., 2020). Interestingly, similar findings on increased reabsorption of BAs have already been observed in healthy animals overexpressing human sEng; however, the exact regulatory signaling activated by sEng is still unclear (Dolezelova et al., 2019). The mechanisms and potential clinical implications of our results are summarized in Figure 7.

In conclusion, we showed here for the first time that labetalol treatment worsens ethinylestradiol-induced intrahepatic cholestasis in mice by increasing BA levels in plasma *via* increased efflux of BA from hepatocytes to plasma through induced Mrp4 transporter. Relative to the increase in BA plasma concentrations, estrogen also increased plasma concentrations of sEng, which was further accentuated by the addition of labetalol. The association of increased BA concentrations with increased plasma sEng levels was verified in pregnant women with ICP, which suggests that sEng may be tested as a novel biomarker of ICP. In addition, transgenic mice with high levels of human sEng showed aggravated estrogen-induced BA accumulation in the plasma of labetalol-treated animals *via* enhanced reabsorption of BAs in the ileum due to the upregulation of the Asbt transporter. It confirms the role of sEng in the regulation of BA homeostasis. Considering the increased coincidence of preeclampsia and ICP, we might propose that labetalol treatment of preeclamptic women may worsen preexisting ICP by increasing plasma BA concentrations. In addition, increased sEng levels in women with ICP justify the fact that these patients are at higher risk of developing preeclampsia than others and explain the potential reason for the increased coincidence of both disorders. Therefore, our results suggest the importance of monitoring sEng and BA concentrations in the plasma of pregnant women that are at high risk of preeclampsia and/or ICP development.

## Data Availability

The original contributions presented in the study are included in the article/Supplementary Material, further inquiries can be directed to the corresponding authors.
