# Supplementary material for: Labetalol and soluble endoglin aggravate bile acid retention in mice with ethinylestradiol-induced cholestasis
Source: Front Pharmacol. 2023 Jan 26;14:1116422. doi: 10.3389/fphar.2023.1116422 (PMC9909014; doi:10.3389/fphar.2023.1116422)
Supplement: Supplementary file 1 [file Presentation1.pdf]

Supplementary Material

Labetalol and soluble endoglin aggravate bile acid retention in mice with ethinylestradiol-induced cholestasis

Ivone Cristina Igreja Sá[1], Katarina Tripska[1], Fatemeh Alaei Faradonbeh[2], Milos Hroch[3], Hana Lastuvkova[2], Jolana Schreiberova[2], Marian Kacerovsky[4], Miguel Pericacho[5], Petr Nachtigal[1*], Stanislav Micuda[2*]

*** Correspondence (**authors contributed equally to the correspondence):

prof. Stanislav Micuda, MD, PhD.: email address: micuda@lfhk.cuni.cz

prof. Petr Nachtigal, PharmD, PhD.: email address: petr.nachtigal@faf.cuni.cz

1 Supplementary Figures and Tables

1.1 Supplementary Figures

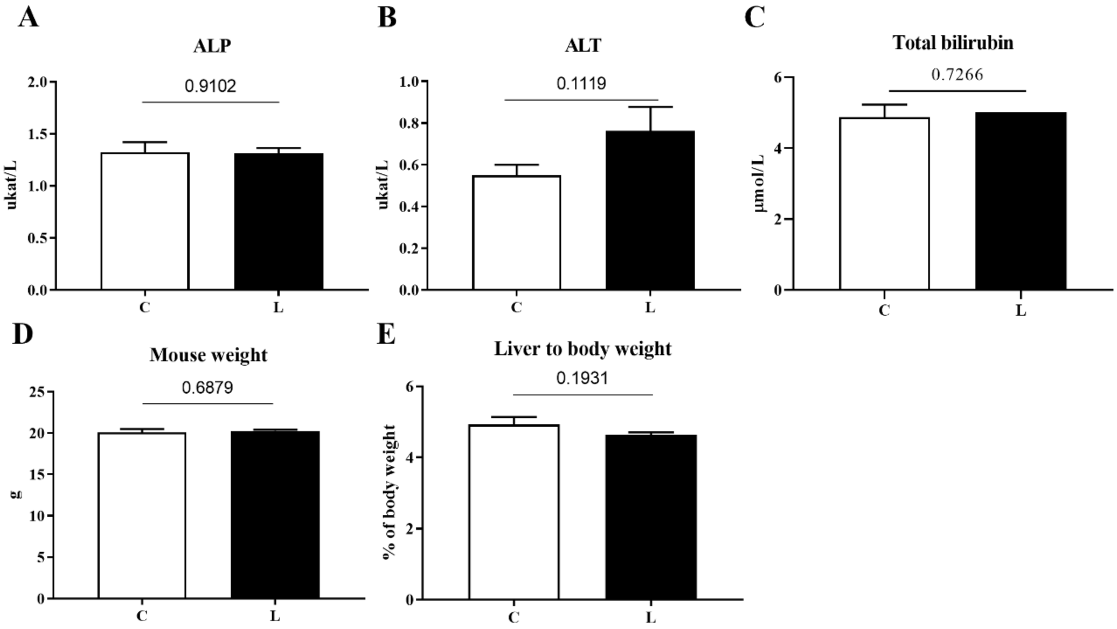

Supplementary Figure 1. Labetalol treatment in healthy animals did not affect body and liver weight, ALP and ALT activity in plasma, or total bilirubin levels. Activity of ALP (A) and ALT (B) and bilirubin levels (C) in plasma. Mouse weight (D) and ratio liver to body weight (E). The data are presented as median with SEM (n=8). Statistical analysis by Unpaired t test. A difference of $P < 0.05$ was considered statistically significant.

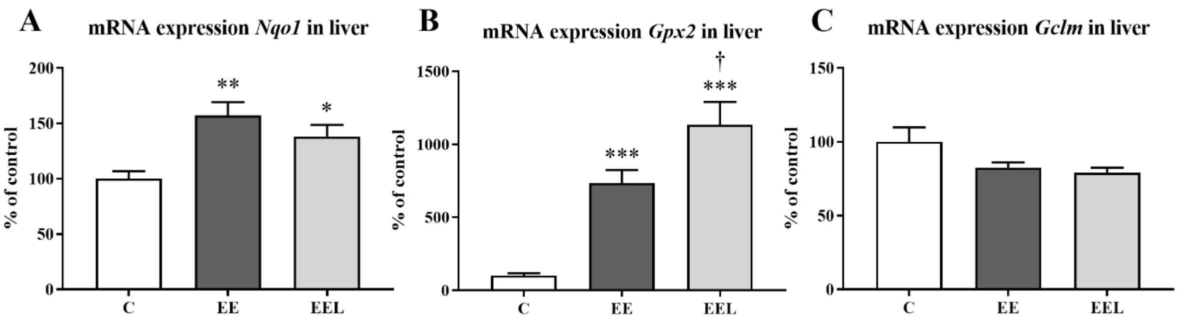

Supplementary Figure 2. The hepatic mRNA expression of Nrf2-target genes. The mRNA expression of *Nqo1 (NAD(P)H quinone dehydrogenase 1)* (A), *Gpx2 (glutathione peroxidase 2)* (B), and *Gclm (glutamate-cysteine ligase modifier subunit)* (C) were measured. The data are presented as median with SEM (n=8). *P<0.05, ***P* < 0.01, ****P* < 0.001, by One-Way ANOVA comparing vehicle-administered control group with cholestatic groups. †P < 0.05 using the unpaired t-test for cholestatic animals (EE) vs. cholestatic animals treated with labetalol (EEL).

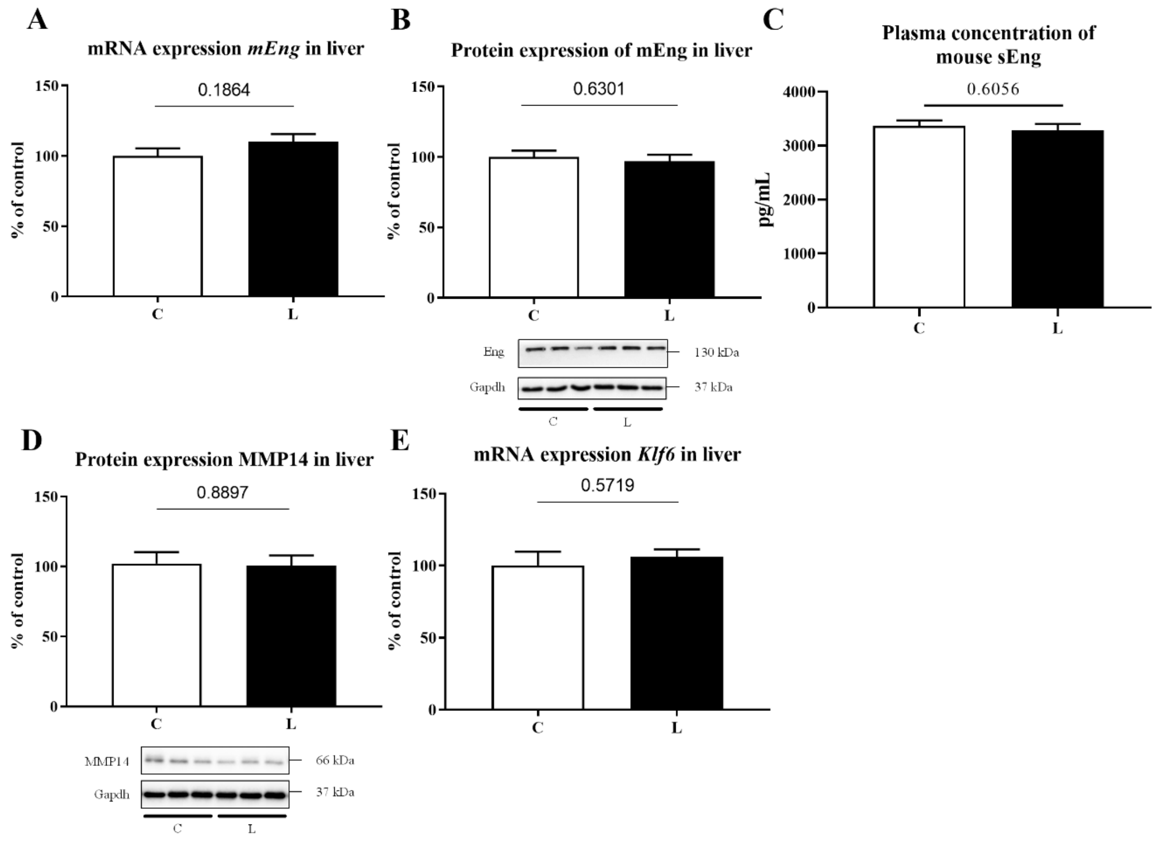

Supplementary Figure 3. Labetalol treatment in healthy animals does not modulate endoglin expression and cleavage. mRNA (A) and protein (B) expression of mouse Eng in liver. Plasma concentration of mouse soluble endoglin (C). Protein expression of MMP14 in liver (D) and mRNA expression of *Klf6* in liver (E). The data are presented as median with SEM (n=8). Statistical analysis by Unpaired t test. A difference of $P < 0.05$ was considered statistically significant.

1.2 Supplementary Tables

Table S1 Pre-designed TaqMan® Gene Expression Assay kits (Life Technologies) used for quantitative real-time RT-PCR

Gene symbol	Life Technologies cat. number
mEng	Mm00468256_m1
Klf6	Mm00516184_m1
Nqo1	Mm01253561_m1
Gpx2	Mm01324400_m1
Gclm	Mm00850074_g1
Gapdh	Mm99999915_g1

Table S2 Primary and secondary antibodies used in Western blot.

Protein	Source	Dilution	Secondary antibody dilution
Ntcp	Thermo Fisher (PA5-80001)	1:1000	1:4000
Mrp4	Cell Signaling (#12857S)	1:2000	1:4000
Bsep	Thermo Fisher (PA5-78690)	1:1000	1:2000
Cyp7a1	Sigma (MABD42)	1:2000	1:5000
Cyp8b1	Thermo Fisher (PA5-37088)	1:1000	1:2000
Cyp27a1	Thermo Fisher (PA5-27946)	1:1000	1:2000
Eng	Santa Cruz (sc19793)	1:500	1:3000
MMP14	Abcam (ab51074)	1:2000	1:4000
Asbt	Thermo Fisher (PA-18990)	1:500	1:2000
Ostα	Biorbyt (orb185685)	1:250	1:2000
Ostβ	Bioss (bs2128R)	1:250	1:2000
Gapdh	Cell Signaling (#2118)	1:8000	1:10000
